# Aortic valve stenosis—multimodality assessment with PET/CT and PET/MRI

**DOI:** 10.1259/bjr.20190688

**Published:** 2019-11-01

**Authors:** Evangelos Tzolos, Jack PM Andrews, Marc R. Dweck

**Affiliations:** 1 British Heart Foundation Centre for Cardiovascular Science, University of Edinburgh, Edinburgh, United Kingdom

## Abstract

Aortic valve disease is the most common form of heart valve disease in developed countries and a growing healthcare burden with an ageing population. Transthoracic and transoesophageal echocardiography remains central to the diagnosis and surveillance of patients with aortic stenosis, providing gold standard assessments of valve haemodynamics and myocardial performance. However, other multimodality imaging techniques are being explored for the assessment of aortic stenosis, including combined PET/CT and PET/MR. Both approaches provide unique information with respect to disease activity in the valve alongside more conventional anatomic assessments of the valve and myocardium in this condition. This review investigates the emerging use of PET/CT and PET/MR to assess patients with aortic stenosis, examining how the complementary data provided by each modality may be used for research applications and potentially in future clinical practice.

## Introduction

Aortic stenosis is the most common valvular heart disease in the developed world. The prevalence of aortic sclerosis in the general population is estimated to be 25% at the age of 65 increasing to almost 50% at age 80. The rate of progression to hemodynamically significant aortic stenosis is estimated to be almost 2% per year, translating to a relatively low prevalence (<1%) at age 60 and younger,^1,2^ but rising rapidly to >10% for elderly patients aged >75.^3^

Aortic stenosis was once thought simply related to age-associated wear and tear but is now known to be an active, highly regulated process with pathophysiological similarities to atherosclerosis and bone formation. Initial endothelial injury leads to lipid and inflammatory cell infiltration followed by the development of progressive valvular fibrosis and calcification. A progressive vicious cycle of calcification and endothelial injury then ensues leading to progressive valve stiffening and stenosis^4^ and consequent left ventricular hypertrophy. Without appropriately timed valve replacement patients end up with symptoms, heart failure and finally death.

In aortic stenosis, imaging of the valve is crucial in ascertaining a diagnosis, grading severity and informing the timing of valvular intervention.^5^ In addition, the importance of the myocardial remodelling response to these forms of valve disease is increasingly appreciated.^6^ While echocardiography remains the gold standard method for assessing patients with aortic stenosis, CT and MR are being used increasingly to provide complimentary information on valve stenosis severity and myocardial health, respectively. Most recently PET has emerged, providing unique information regarding disease activity in combination with either CT or MR assessments.

In this review, we will focus on how modern advances in PET/CT and PET/MR might improve our pathophysiological understanding of aortic stenosis, aid in the development of novel treatment strategies and ultimately improve the care of patients with aortic stenosis.

### How does PET work?

**Figure 1.**
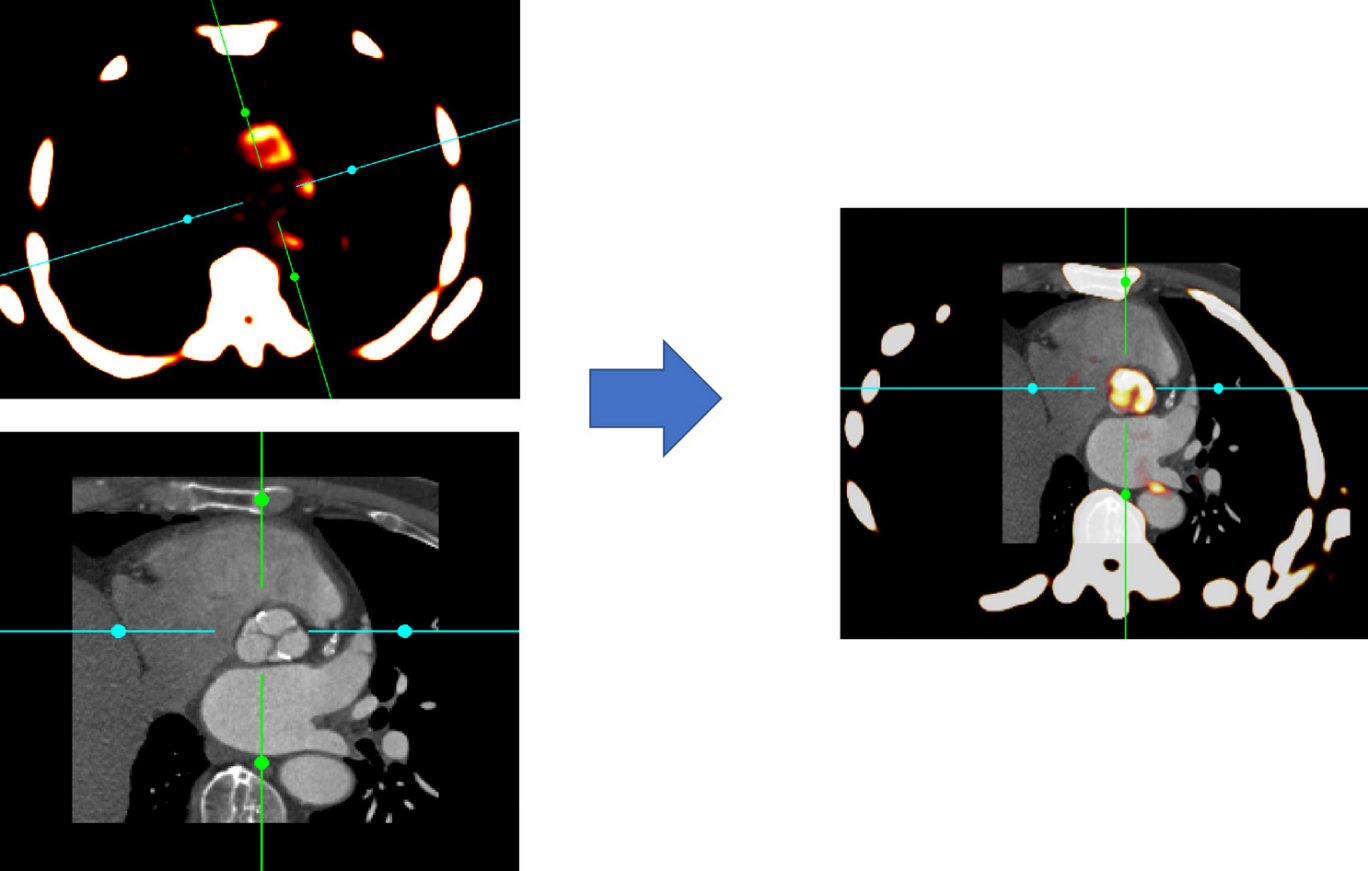
Basis of PET/CT imaging. PET/CT scanners incorporate functional data from PET and anatomical information from CT imaging on the same gantry allowing near simultaneous acquisition. Fused PET/CT images then allow localization of specific pathological processes to individual structures such as the aortic valve in this example. (Images reconstructed using FusionQuant)

Modern hybrid PET/CT and PET/MR scanners now provide detailed molecular information about the activity of specific disease processes developing *in vivo*. In principle any biologically active process can be studied depending on the availability of a targeted PET radiotracer. These radiotracers are injected into the body and accumulate in areas of active disease, emitting radiation that can be detected by the PET scanner to create a PET image. This image lack spatial resolution but this is overcome by fusion with an anatomical image provided by either CT or MR, captured with the patient lying in the same position on a single gantry (Figure 1). These scans also offer attenuation correction allowing quantification of tracer concentration in different tissues. Additional CT and MRIsequences now allow combination of the disease activity information from PET with advanced structural information from CT or MR, harnessing all the advantages of these advanced imaging techniques.^7^

#### Developing role of CT and MR in calcific AS

Transthoracic echocardiography (TTE) is generally sufficient enough to assess the aortic valve morphology (tricuspid *vs* bicuspid) and the haemodynamic assessment of the valve. However, in around a quarter of patients with moderate of severe aortic stenosis, echocardiographic assessments of disease severity are discordant resulting in diagnostic uncertainty. Recently, multidetector CT (MDCT) calcium scoring of the valve (CT-AVC) has emerged as an alternative assessment of aortic stenosis severity to complement echocardiography. CT-AVC provides a quantitative and accurate measurement of aortic valve calcium (AVC) burden, with high intraobserver, interobserver and scan–rescan reproducibility.^8,9^ Sex-specific thresholds for severe disease demonstrate excellent agreement with concordant echocardiography (AUC 0.92 in female and 0.89 in male Pawade et al)^10^ and importantly provide strong prediction of disease progression and the need for AVR compared to echocardiography.^11–17^ On this basis, CT-AVC was included in the most recent European Society of Cardiology guidelines for the assessment of patients with discordant echocardiographic measurements.^17^ Contrast-enhanced CT angiography is now used as standard in the work up of patients being considered for transcutaneous aortic valve implantation, providing accurate valve sizing, distance to the coronary arteries and assessment of the optimal access route (ref).

Cardiac MR (CMR) can also be used in cases where echocardiographic results are inconclusive. It has multiple advantages, including the ability to measure heart volumes, blood flow and ventricular wall thickness.^18–21^ CMR is a useful tool in the diagnosis and evaluation of bicuspid aortic valve and is invaluable in the assessment of concomitant thoracic aortic dilatation/aneurysm and mitral valve abnormalities.^22,23^ However, the real strength of CMR lies in the assessment of the myocardium and in particular of myocardial fibrosis.^24–26^ Multiple studies have now shown that midwall fibrosis (as detected through late gadolinium enhancement) serves as an objective marker of left ventricular decompensation and is an independent predictor of mortality in patients with aortic stenosis.^25,27–29^ The EVOLVED study (Early Valve Replacement Guided by Biomarkers of LV Decompensation in Asymptomatic Patients with Severe Aortic Stenosis, NCT03094143) aims to assess the clinical outcome of asymptomatic patients with severe aortic stenosis and mid-wall myocardial fibrosis that have either early surgical intervention or routine care.

### Positron Emission Tomography

Hybrid imaging platforms such as PET/CT and PET/MR can now incorporate the benefits of both anatomical (CT and MR) and molecular (PET) techniques to provide a comprehensive imaging assessment of aortic stenosis. In particular, the PET component provides assessments of disease activity to complement the anatomic information provided by the other modalities. Recently, PET has utilised two tracers: ^18^F-fluorodeoxyglucose (^18^F-FDG) to measure inflammation in the valves of patients with aortic stenosis and ^18^F-fluoride for calcification activity.

^18^F-FDG PET is widely used to image vascular inflammation. This PET tracer is an inactive glucose analogue which concentrates in metabolically active cells and is a well-established, sensitive but non-specific marker of vascular inflammation. Macrophages have higher glucose requirements than surrounding cells in the vasculature, which translates in excellent correlation (*r* = 0.85, *p* < 0.001) between the ^18^F-FDG signal and macrophage burden on histology (CD68 staining) in carotid atheroma.^30^

18F-fluoride has been used both clinical and in oncological research for over 50 years as a bone tracer. Fluoride ions exchange with hydroxyl ions in areas of exposed hydroxyapatite crystal, with binding highest in areas of developing microcalcification due to surface area effects.^31^ In preferentially targeting developing microcalcification, 18F-Fluoride PET therefore provides different information to the macrocalcification identified by CT.

### PET/CT

18F-FDG PET/CT imaging in aortic stenosis was first explored by Marincheva-Sancheva in 2011.^32^ In a retrospective observational study, 84 patients (42 with aortic stenosis and 42 age-matched control patients without aortic stenosis) were recruited from a list of subjects who endured PET/CT imaging between 2005 and 2010, primarily for assessment of cancer. Severity of aortic stenosis was established on echocardiography and patients were respectively divided in three groups (mild, moderate and severe aortic stenosis). Overall the AV PET signal, mean Target to Background Ratio (TBR_mean_), was higher in patients with aortic stenosis compared to controls: median 1.53 (IQR: 1.42 to 1.76) *vs* 1.34 (IQR: 1.20 to 1.55); *p* < 0.001. Interestingly on further assessment, 18F-FDG uptake was higher in patients with mild and moderate aortic stenosis than controls but was no different in those with severe AS. Comparable tendencies were detected when subjects were classified according to AV calcification. Furthermore, compared with the 18F-FDG uptake in non-calcified AVs, TBR was increased in mildly and moderately, but not severely calcified valves. As predicted, there was a significant association between valvular calcification grade and aortic stenosis severity (*r* = 0.90, *p* < 0.001). Moreover, in a subset of patients, it was observed that the valvular TBR is increased in patients who afterwards experience progression of aortic stenosis. Patients with high AV TBR had a higher probability of haemodynamic progression of aortic stenosis on repeat echo, performed 1 to 2 years after the baseline echo. Notably, progression of aortic stenosis was noted in five of six patients (83%) with high initial AV TBR and only in two of nine patients (22%) with low TBR (*p* = 0.04).

This was the first study to demonstrate that 18F-FDG uptake is increased in aortic stenosis, supporting the hypothesis that it is an active inflammatory condition.

Our group has also confirmed the excellent reproducibility and feasibility of PET/CT in the assessment of aortic stenosis, with 18F-FDG and particularly 18F-NaF exhibiting significant potential as novel biomarkers of aortic stenosis progression.^33^ In a prospective study, 101 successive patients aged >50 years with different stage of aortic valve disease, ranging from aortic sclerosis to severe aortic stenosis, and 20 age-matched control subjects, had 18F-NaF and 18F-FDG scans less than 1 month apart. 18F-NaF uptake strongly correlated with the stage of the aortic valve disease, with patients with aortic stenosis demonstrating the highest uptake followed by the aortic sclerosis and control subjects (TBR_MAX_: *vs* 2.87 ± 0 *vs* 1.92 ± 0.3182 *vs* 1.55 ± 0.17, respectively; *p* < 0.001). In the severe aortic stenosis group (*n* = 23), 100% of subjects had increased uptake compared to 88% in the mild and moderate groups (*n* = 55) and 45% in the aortic sclerosis cohort (*n* = 20).

Although 18F-FDG also showed increased uptake in the valve, a more scattered pattern of activity was observed. We observed similar trends of activity with aortic stenosis demonstrating substantially higher uptake compared with both aortic sclerosis and control subjects. (TBR_MAX_: 1.58 ± 0.21 *vs* 1.47 ± 0.15 *vs* 1.30 ± 0.13, respectively; *p* < 0.001. Compared to 18F-NaF, the correlation of the uptake with the stage of the aortic stenosis severity was weaker, with only 52% (*vs* 100% with 18F-NaF) at the severe aortic stenosis group demonstrating increased uptake. Similarly, just below one-third (31%) in the mild and moderate aortic stenosis showed increased uptake and just one-fourth (20%) in the sclerosis group. Interestingly, while 18F-NaF uptake was higher than 18F-FDG uptake in the valve, the reverse was true in areas of atheroma, highlighting importance pathological differences in these two conditions that might justify the disappointing results of statin therapy in aortic stenosis and indicating that therapies targeting calcification directly might prove of greater clinical value.^34^

A subsequent follow-up study of this cohort demonstrated that both ^18^F-fluoride and ^18^F-FDG predicted disease progression and adverse clinical outcomes in aortic stenosis.^35^ Disease progression was assessed at 1 and 2 years using AVC scoring and TTE. On repeat CT imaging, new valvular calcium was visible in a similar distribution as the ^18^F-fluoride activity on baseline PET imaging. Indeed, baseline ^18^F-fluoride uptake correlated strongly with the subsequent rate of progression in AVC (Spearman *r* = 0.80; *p* < 0.001) and with echocardiographic measures of haemodynamic progression (mean gradient *r* = 0.32; *p* = 0.001). 18F-FDG-PET showed similar but weaker correlations. (*r* = 0.43; *p* < 0.001 and *r* = 0.26; *p* = 0.01, respectively). The study also looked at clinical outcomes. The primary clinical outcome endpoint was a composite of cardiovascular death and aortic valve replacement. Average follow-up was 1232 days with 23 patients (17%) having undergone AVR and nine cardiovascular death.^7^ Both ^18^F-Fluoride and ^18^F-FDG were independent predictors of all-cause mortality and AVR [HR 1.46 (1.24–1.71); *p* < 0.001 and HR 1.59 (1.21–2.09); *p* = 0.002, respectively].

More recent studies have used 18F-NaF to gain insights into the pathophysiology underlying aortic stenosis. Knag et al^36^ showed that PET/CT has a promising role in research studies exploring the pathophysiology of aortic stenosis and finding potential medical therapy targets. They showed that in patients with aortic stenosis, lipoprotein(a)[Lp(a)] and oxidised phospholipids (OxPL), drive valve calcification and disease progression. On baseline PET/CT, patients in the top Lp(a) tertile had increased valve calcification activity and increased 18F-NAF uptake, compared with those in lower tertiles. These findings suggest lowering Lp(a) or inactivating OxPL may slow aortic stenosis progression and provide a rationale for clinical trials to test this hypothesis.^36^

In addition, 18F-NaF PET/CT has been extended to investigate bioprosthetic valve calcification and degeneration. Indeed 18F-NaF appears to provide a highly sensitive marker of early bioprosthetic valve degeneration correlating well with tissue degeneration on histology and providing a powerful prediction tool of subsequent valvular dysfunction and failure of incremental value to standard approaches.^37^ (18F-Fluoride Assessment of Aortic Bioprosthesis Durability and Outcome [18F-FAABULOUS]; NCT02304276)

In summary, PET/CT imaging provides unique information with respect to disease activity in aortic stenosis. It provides powerful prediction of disease progression in both native and prosthetic valve, excellent scan–rescan reproducibility for tracer quantification (refs) as well as important biological insights. On this basis, it is being used as a marker of disease activity and efficacy endpoint in several ongoing trials of novel therapies in aortic stenosis. In SALTIRE 2 (NCT02132026) 75 subjects will be randomised (2:1) to either subcutaneous denosumab 60 mg (*n* = 50) or matched placebo (*n* = 25) every 6 months; and a further 75 will be randomised (2:1) to oral alendronate 70 mg (*n* = 50) or matched placebo (*n* = 25) once week. 18F-NaF uptake will be measured at baseline, and 6 months to assess the early impact of the intervention on valvular calcification activity. Its future role in clinical practice is less clear, given that the simpler technique of CT calcium scoring also provides similar prediction of disease progression and clinical events. However, if advances in the PET/CT technique including motion correction and co-registration result in improvements such that PET outperforms in CT, then this approach may yet develop a clinical role, with initial results suggesting that this may occur sooner in bioprosthetic rather than native valves.

### PET/MR

PET/MR is an emerging technique, combining the excellent temporal and spatial resolution of MRI with the sensitive molecular imaging of PET. In principle, PET/MR offers several potential advantages over PET/CT, in particular superior tissue characterisation, improved motion correction and major reductions in radiation exposure. Furthermore, MR is also more naturally suited to the imaging of certain tissues in the body compared to CT including the left ventricular myocardium. However, the availability of PET/MR is currently limited, and scanners remain expensive. Moreover, several technical challenges remain with cardiac PET/MR scanning as outlined below.

First, the method of PET attenuation correction is different between PET/CT and PET/MR. Unlike CT, MR attenuation correction maps are based on proton density,^38^ with two current approaches. The Dixon MR AC map is the standard approach but is hampered in cardiac studies due to both motion artefact (along the heart-lung and liver-lung interfaces) and mis-segmentation of the bronchi as soft tissue. A novel free-breathing radial Gradient Recalled Echo approach has been demonstrated in patients with coronary disease to largely overcome these issues^39^ but concerns regarding signal dropout in those with metallic implants such as aortic valve replacements and stents remain.

Second, both cardiac and respiratory motion causes loss of PET signal which subsequently reduces reader confidence in image interpretability. In principle, PET/MR might allow the tracking of motion throughout PET acquisition and therefore its correction. In practice, this has yet to be consistently applied. Finally, PET/MR is a costly technique which requires considerable expertise within centres for both performing and interpreting scans, rendering both research output and clinical adoption slow when compared to PET/CT.

#### PET/MR of the aortic valve

**Figure 2.**
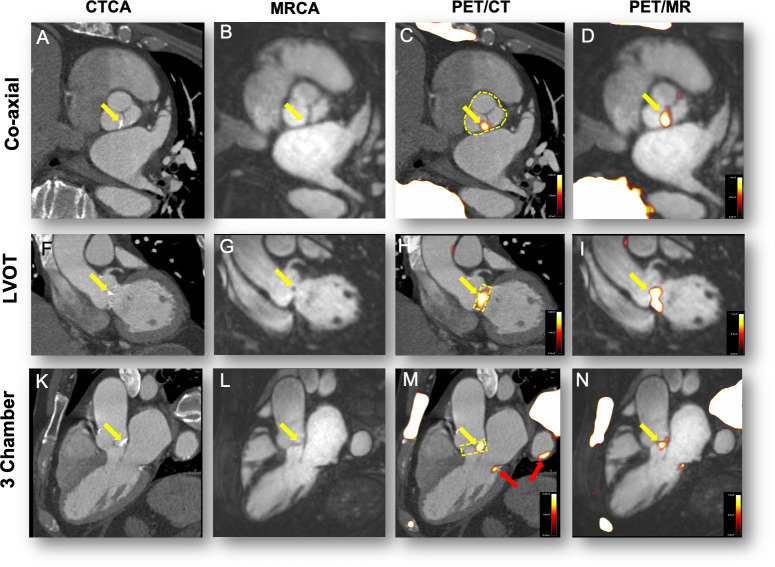
^18^F-fluoride uptake in a patient with moderate aortic stenosis. The columns represent the imaging modality and rows the corresponding view. Panels A, F and G show calcification of the aortic valve (non-coronary cusp predominantly, yellow arrows). Panels B, G and L show the CMRA in the same views. Calcification cannot be appreciated on MR but the raphe between the NCC and LCC appears thickened (B). PET/CT shows uptake overlaying these areas of calcification (Panels C, (H and M). Note uptake also over the calcified mitral annulus (M, red arrow) and arterial wall of the descending aorta (M, red arrow). Fused PET/MR shows ^18^F-fluoride uptake in the same areas as the PET/CT (D, I and N).

Despite these limitation PET/MR has demonstrated some early promise in the assessment of patients with aortic stenosis. Doris et al were the first to report increased 18F-fluoride activity on PET/MR in aortic stenosis (Figure 2) and also illustrated the versatility of the technique providing assessments of LV volumes, mass, ejection fraction and LGE burden alongside the valve assessments. Moreover, they demonstrated improved signal-to-noise ratios (SNR) and TBRs over the valve using motion correction approaches derived from the PET.^40^ Further studies are now required.

Outside the aortic valve, both 18F-FDG and 18F-fluoride PET/MR have been shown to delineate areas of infarcted myocardium.^41,42^ In inflammatory disease, 18F-FDG PET/MR has demonstrated major promise in the clinical diagnosis of active cardiac sarcoid combining the advantages of PET and MR LGE.^43,44^ This approach has also shown promise in identifying areas of florid inflammation in myocarditis and endocarditis.^45,46^ Furthermore, 18F-fluoride PET/MR may help differentiate between different subtypes of cardiac amyloid.^47^ Given the increasing prevalence (and similar phenotypes) of both aortic stenosis and cardiac amyloid in an ageing population, our group is currently utilising PET/MR to investigate whether 18F-fluoride can distinguish clinically significant aortic stenosis from cardiac amyloid as well as comparing uptake in healthy controls (NCT03352089).

PET/MR holds particular promise in the temporal assessment of chronic slowly progressing conditions such as bicuspid aortic valve disease which is present in 1.4% of live births and contributes significantly to the proportion of patients undergoing aortic valve surgery.^48–50^ The greatly reduced radiation exposure is particularly important given the relatively younger age of the patient at presentation and indeed this is why ^18^F-Fluoride PET/MR is being explored as an efficacy endpoint in the BASIK two trial (Bicuspid Aortic Valve Stenosis and the Effect of Vitamin K2 on Calcification Using ^18^F -Sodium Fluoride Positron Emission Tomography/Magnetic Resonance**,** NCT02917525).^51^ Furthermore, the reduced radiation dose afforded through PET/MR may allow the exploration of multiple tracers used simultaneously to enhance our understanding of the temporal relationship between inflammation, fibrosis and calcification within the disease process. However, before PET/MR can be widely applied to cardiovascular disease, it is important to establish whether PET/MR provides similar results to PET/CT. We have therefore recently completed a simultaneous PET/CT and PET/MR validation study in patients with coronary artery disease and aortic stenosis which will report soon (NCT02988531). Moreover, the BASIK2 trial (NCT02917525) is now being conducted to investigate the effect of vitamin K2 supplementation on valvular calcification in patients with bicuspid aortic valve disease.^51^ The primary endpoint is the change in PET/MR 18F-NaF uptake (6 months minus baseline).

## Conclusion

PET/CT can be used to measure inflammation and valvular calcification activity in patients with aortic stenosis, providing important insights into the pathogenesis of aortic stenosis and a useful surrogate endpoint of disease activity in trials of novel therapies. Moreover ^18^F-Fluoride PET provides powerful prediction of disease progression and adverse events, with further development required to demonstrate incremental value to CT calcium scoring. Despite its inferior spatial resolution, PET/MR can provide detailed assessment of both valvular function and hypertrophic response with a lower associated radiation exposure. While in the early stages of its development, PET/MR may emerge as the favoured technique for temporal disease tracking and myocardial assessments. Further research is now warranted using both these exciting techniques to imaging patients with aortic stenosis.

## References

[1] Nkomo VT, Gardin JM, Skelton TN, Gottdiener JS, Scott CG, Enriquez-Sarano M. Burden of valvular heart diseases: a population-based study. The Lancet 2006; 368: 1005–11. doi: 10.1016/S0140-6736(06)69208-8 16980116

[2] Eveborn GW, Schirmer H, Heggelund G, Lunde P, Rasmussen K. The evolving epidemiology of valvular aortic stenosis. The Tromsø study. Heart 2013; 99: 396–400. doi: 10.1136/heartjnl-2012-302265 22942293

[3] Osnabrugge RL, Mylotte D, Head SJ, Van Mieghem NM, Nkomo VT, LeReun CM, et al. Aortic stenosis in the elderly: disease prevalence and number of candidates for transcatheter aortic valve replacement: a meta-analysis and modeling study. J Am Coll Cardiol 2013; 62: 1002–12. 23727214 10.1016/j.jacc.2013.05.015

[4] Pawade TA, Newby DE, Dweck MR. Calcification in aortic stenosis: the skeleton key. J Am Coll Cardiol 2015; 66: 561–77. 26227196 10.1016/j.jacc.2015.05.066

[5] Everett RJ, Newby DE, Jabbour A, Fayad ZA, Dweck MR. The role of imaging in aortic valve disease. Curr Cardiovasc Imaging Rep 2016; 9: 21. doi: 10.1007/s12410-016-9383-z 27375833 PMC4896976

[6] Dweck MR, Boon NA, Newby DE. Calcific aortic stenosis: a disease of the valve and the myocardium. J Am Coll Cardiol 2012; 60: 1854–63. 23062541 10.1016/j.jacc.2012.02.093

[7] Bockisch A, Beyer T, Antoch G, Freudenberg L, Kuhl H, Debatin J, et al. Positron emission tomography/computed tomography?imaging protocols, artifacts, and pitfalls. Molecular Imaging & Biology 2004; 6: 188–99. doi: 10.1016/j.mibio.2004.04.006 15262234

[8] Messika-Zeitoun D, Aubry M-C, Detaint D, Bielak LF, Peyser PA, Sheedy PF, et al. Evaluation and clinical implications of aortic valve calcification measured by electron-beam computed tomography. Circulation 2004; 110: 356–62. doi: 10.1161/01.CIR.0000135469.82545.D0 15249504

[9] Cowell SJ, Newby DE, Burton J, White A, Northridge DB, Boon NA, et al. Aortic valve calcification on computed tomography predicts the severity of aortic stenosis. Clin Radiol 2003; 58: 712–6. doi: 10.1016/S0009-9260(03)00184-3 12943644

[10] Pawade T, Clavel M-A, Tribouilloy C, Dreyfus J, Mathieu T, Tastet L, et al. Computed tomography aortic valve calcium scoring in patients with aortic stenosis. Circulation 2018; 11: e007146. doi: 10.1161/CIRCIMAGING.117.007146 29555836

[11] Messika-Zeitoun D, Bielak LF, Peyser PA, Sheedy PF, Turner ST, Nkomo VT, et al. Aortic valve calcification: determinants and progression in the population. Arterioscler Thromb Vasc Biol 2007; 27: 642–8. 17185617 10.1161/01.ATV.0000255952.47980.c2

[12] Nguyen V, Cimadevilla C, Estellat C, Codogno I, Huart V, Benessiano J, et al. Haemodynamic and anatomic progression of aortic stenosis. Heart 2015; 101: 943–7. doi: 10.1136/heartjnl-2014-307154 25655063

[13] Dweck MR, Jenkins WSA, Vesey AT, Pringle MAH, Chin CWL, Malley TS, et al. 18F-sodium fluoride uptake is a marker of active calcification and disease progression in patients with aortic stenosis. Circulation 2014; 7: 371–8. doi: 10.1161/CIRCIMAGING.113.001508 24508669

[14] Cueff C, Serfaty J-M, Cimadevilla C, Laissy J-P, Himbert D, Tubach F, et al. Measurement of aortic valve calcification using multislice computed tomography: correlation with haemodynamic severity of aortic stenosis and clinical implication for patients with low ejection fraction. Heart 2011; 97: 721–6. doi: 10.1136/hrt.2010.198853 20720250

[15] Clavel MA, Messika-Zeitoun D, Pibarot P, Aggarwal SR, Malouf J, Araoz PA, et al. The complex nature of discordant severe calcified aortic valve disease grading: new insights from combined Doppler echocardiographic and computed tomographic study. J Am Coll Cardiol 2013; 62: 2329–38. 24076528 10.1016/j.jacc.2013.08.1621

[16] Clavel MA, Pibarot P, Messika-Zeitoun D, Capoulade R, Malouf J, Aggarval S, et al. Impact of aortic valve calcification, as measured by MDCT, on survival in patients with aortic stenosis: results of an international registry study. J Am Coll Cardiol 2014; 64: 1202–13. 25236511 10.1016/j.jacc.2014.05.066 PMC4391203

[17] Baumgartner H, Falk V, Bax JJ, De Bonis M, Hamm C, Holm PJ, et al. 2017 ESC/EACTS guidelines for the management of valvular heart disease. Eur Heart J 2017; 38: 2739–91. doi: 10.1093/eurheartj/ehx391 28886619

[18] Dweck MR, Joshi S, Murigu T, Gulati A, Alpendurada F, Jabbour A, et al. Left ventricular remodeling and hypertrophy in patients with aortic stenosis: insights from cardiovascular magnetic resonance. J Cardiovasc Magn Reson 2012; 14: 50. doi: 10.1186/1532-429X-14-50 22839417 PMC3457907

[19] Gunther S, Grossman W. Determinants of ventricular function in pressure-overload hypertrophy in man. Circulation 1979; 59: 679–88. doi: 10.1161/01.CIR.59.4.679 154367

[20] Salcedo EE, Korzick DH, Currie PJ, Stewart WJ, Lever HM, Goormastic M. Determinants of left ventricular hypertrophy in patients with aortic stenosis. Cleve Clin J Med 1989; 56: 590–6. doi: 10.3949/ccjm.56.6.590 2530009

[21] Hein S, Arnon E, Kostin S, Schonburg M, Elsasser A, Polyakova V, et al. Progression from compensated hypertrophy to failure in the pressure-overloaded human heart: structural deterioration and compensatory mechanisms. Circulation 2003; 107: 984–91. 12600911 10.1161/01.cir.0000051865.66123.b7

[22] Gleeson TG, Mwangi I, Horgan SJ, Cradock A, Fitzpatrick P, Murray JG, et al. SSFP) cine MRI in distinguishing normal and bicuspid aortic valves. J Magn Reson Imaging 2008; 28: 873–8. 18821622 10.1002/jmri.21547

[23] Rajiah P. Ct and MRI in the evaluation of thoracic aortic diseases. Int J Vasc Med 2013; 2013: 1–16. doi: 10.1155/2013/797189 PMC3874367 24396601

[24] Rudolph A, Abdel-Aty H, Bohl S, Boyé P, Zagrosek A, Dietz R, et al. Noninvasive detection of fibrosis applying contrast-enhanced cardiac magnetic resonance in different forms of left ventricular hypertrophy. J Am Coll Cardiol 2009; 53: 284–91. doi: 10.1016/j.jacc.2008.08.064 19147047

[25] Dweck MR, Joshi S, Murigu T, Alpendurada F, Jabbour A, Melina G, et al. Midwall fibrosis is an independent predictor of mortality in patients with aortic stenosis. J Am Coll Cardiol 2011; 58: 1271–9. doi: 10.1016/j.jacc.2011.03.064 21903062

[26] Barone-Rochette G, Piérard S, De Meester de Ravenstein C, Seldrum S, Melchior J, Maes F, et al. Prognostic significance of LGE by CMR in aortic stenosis patients undergoing valve replacement. J Am Coll Cardiol 2014; 64: 144–54. doi: 10.1016/j.jacc.2014.02.612 25011718

[27] Kwong RY, Chan AK, Brown KA, Chan CW, Reynolds HG, Tsang S, et al. Impact of unrecognized myocardial scar detected by cardiac magnetic resonance imaging on event-free survival in patients presenting with signs or symptoms of coronary artery disease. Circulation 2006; 113: 2733–43. doi: 10.1161/CIRCULATIONAHA.105.570648 16754804

[28] Assomull RG, Prasad SK, Lyne J, Smith G, Burman ED, Khan M, et al. Cardiovascular magnetic resonance, fibrosis, and prognosis in dilated cardiomyopathy. J Am Coll Cardiol 2006; 48: 1977–85. doi: 10.1016/j.jacc.2006.07.049 17112987

[29] Vahanian A, Otto CM. Risk stratification of patients with aortic stenosis. Eur Heart J 2010; 31: 416–23. doi: 10.1093/eurheartj/ehp575 20047994

[30] Tawakol A, Migrino RQ, Bashian GG, Bedri S, Vermylen D, Cury RC, et al. In vivo 18F-fluorodeoxyglucose positron emission tomography imaging provides a noninvasive measure of carotid plaque inflammation in patients. J Am Coll Cardiol 2006; 48: 1818–24. doi: 10.1016/j.jacc.2006.05.076 17084256

[31] Irkle A, Vesey AT, Lewis DY, Skepper JN, Bird JLE, Dweck MR, et al. Identifying active vascular microcalcification by 18F-sodium fluoride positron emission tomography. Nat Commun 2015; 6: 7495. doi: 10.1038/ncomms8495 26151378 PMC4506997

[32] Marincheva-Savcheva G, Subramanian S, Qadir S, Figueroa A, Truong Q, Vijayakumar J, et al. Imaging of the aortic valve using fluorodeoxyglucose positron emission tomography increased valvular fluorodeoxyglucose uptake in aortic stenosis. J Am Coll Cardiol 2011; 57: 2507–15. 21679852 10.1016/j.jacc.2010.12.046

[33] Dweck MR, Jones C, Joshi NV, Fletcher AM, Richardson H, White A, et al. Assessment of valvular calcification and inflammation by positron emission tomography in patients with aortic stenosis. Circulation 2012; 125: 76–86. doi: 10.1161/CIRCULATIONAHA.111.051052 22090163

[34] Dweck MR, Khaw HJ, Sng GKZ, Luo ELC, Baird A, Williams MC, et al. Aortic stenosis, atherosclerosis, and skeletal bone: is there a common link with calcification and inflammation? Eur Heart J 2013; 34: 1567–74. doi: 10.1093/eurheartj/eht034 23391586

[35] Jenkins WSA, Vesey AT, Shah ASV, Pawade TA, Chin CWL, White AC, et al. Valvular 18F-Fluoride and 18F-fluorodeoxyglucose uptake predict disease progression and clinical outcome in patients with aortic stenosis. J Am Coll Cardiol 2015; 66: 1200–1. doi: 10.1016/j.jacc.2015.06.1325 26338001

[36] Zheng KH, Tsimikas S, Pawade T, Kroon J, Jenkins WSA, Doris MK, et al. Lipoprotein(a) and Oxidized Phospholipids Promote Valve Calcification in Patients With Aortic Stenosis. J Am Coll Cardiol 2019; 73: 2150–62. doi: 10.1016/j.jacc.2019.01.070 31047003 PMC6494952

[37] Cartlidge TRG, Doris MK, Sellers SL, Pawade TA, White AC, Pessotto R, et al. Detection and Prediction of Bioprosthetic Aortic Valve Degeneration. J Am Coll Cardiol 2019; 73: 1107–19. doi: 10.1016/j.jacc.2018.12.056 30871693 PMC6424589

[38] Beyer T, Lassen ML, Boellaard R, Delso G, Yaqub M, Sattler B, et al. Investigating the state-of-the-art in whole-body MR-based attenuation correction: an intra-individual, inter-system, inventory study on three clinical PET/MR systems. Magn Reson Mater Phy 2016; 29: 75–87. doi: 10.1007/s10334-015-0505-4 26739263

[39] Robson PM, Dweck MR, Trivieri MG, Abgral R, Karakatsanis NA, Contreras J, et al. Coronary artery PET/MR imaging: feasibility, limitations, and solutions. JACC Cardiovasc Imaging 2017; 10(10 Pt A): 1103–12. 28109921 10.1016/j.jcmg.2016.09.029 PMC5509532

[40] Doris MK, Rubeaux M, Pawade T, Otaki Y, Xie Y, Li D, et al. Motion-Corrected imaging of the aortic valve with. J Nucl Med 2017; 58: 1811–4. 28546334 10.2967/jnumed.117.194597

[41] Marchesseau S, Seneviratna A, Sjöholm AT, Qin DL, JXM H, Hausenloy DJ, et al. Hybrid PET/CT and PET/MRI imaging of vulnerable coronary plaque and myocardial scar tissue in acute myocardial infarction. J Nucl Cardiol 2017;. 10.1007/s12350-017-0918-8 28500539

[42] Rischpler C, Langwieser N, Souvatzoglou M, Batrice A, van Marwick S, Snajberk J, et al. Pet/Mri early after myocardial infarction: evaluation of viability with late gadolinium enhancement transmurality vs. 18F-FDG uptake. Eur Heart J Cardiovasc Imaging 2015; 16: 661–9. 25680385 10.1093/ehjci/jeu317

[43] Abgral R, Dweck MR, Trivieri MG, Robson PM, Karakatsanis N, Mani V, et al. Clinical utility of combined FDG-PET/MR to assess myocardial disease. JACC: Cardiovascular Imaging 2017; 10: 594–7. doi: 10.1016/j.jcmg.2016.02.029 27372018 PMC5199624

[44] Dweck MR, Abgral R, Trivieri MG, Robson PM, Karakatsanis N, Mani V, et al. Hybrid Magnetic Resonance Imaging and Positron Emission Tomography With Fluorodeoxyglucose to Diagnose Active Cardiac Sarcoidosis. JACC: Cardiovascular Imaging 2018; 11: 94–107. doi: 10.1016/j.jcmg.2017.02.021 28624396 PMC5995315

[45] Nensa F, Poeppel TD, Krings P, Schlosser T. Multiparametric assessment of myocarditis using simultaneous positron emission tomography/magnetic resonance imaging. Eur Heart J 2014; 35: 2173. doi: 10.1093/eurheartj/ehu086 24578391

[46] Nensa F, Tezgah E, Poeppel TD, Jensen CJ, Schelhorn J, Köhler J, et al. Integrated 18F-FDG PET/MR imaging in the assessment of cardiac masses: a pilot study. Journal of Nuclear Medicine 2015; 56: 255–60. doi: 10.2967/jnumed.114.147744 25552667

[47] Trivieri MG, Dweck MR, Abgral R, Robson PM, Karakatsanis NA, Lala A, et al. 18 F-Sodium Fluoride PET/MR for the Assessment of Cardiac Amyloidosis. J Am Coll Cardiol 2016; 68: 2712–4. doi: 10.1016/j.jacc.2016.09.953 27978955 PMC5438164

[48] Hoffman JIE, Kaplan S. The incidence of congenital heart disease. J Am Coll Cardiol 2002; 39: 1890–900. doi: 10.1016/S0735-1097(02)01886-7 12084585

[49] Hoffman JIE, Kaplan S, Liberthson RR. Prevalence of congenital heart disease. Am Heart J 2004; 147: 425–39. doi: 10.1016/j.ahj.2003.05.003 14999190

[50] Masri A, Svensson LG, Griffin BP, Desai MY. Contemporary natural history of bicuspid aortic valve disease: a systematic review. Heart 2017; 103: 1323–30. doi: 10.1136/heartjnl-2016-309916 28490615

[51] Peeters F, van Mourik MJW, Meex SJR, Bucerius J, Schalla SM, Gerretsen SC, et al. Bicuspid aortic valve stenosis and the effect of vitamin K2 on calcification using. Nutrients 2018; 10. 10.3390/nu10040386 PMC5946171 29561783

